# Supplementary material for: Scorpion Venom Heat-Resistant Synthetic Peptide Alleviates Neuronal Necroptosis in Alzheimer’s Disease Model by Regulating Lnc Gm6410 Under PM2.5 Exposure
Source: Int J Mol Sci. 2025 May 4;26(9):4372. doi: 10.3390/ijms26094372 (PMC12072906; doi:10.3390/ijms26094372)
Supplement: Supplementary file 1 [file ijms-26-04372-s001.zip › ijms-3579399-supplementary-1.pdf]

Supplementary Material

SVHRSP alleviates neuronal necroptosis in the Alzheimer's disease model by regulating Lnc Gm6410 under $PM_{2.5}$ exposure

Chuhao Qin [a], Dongsheng Li [a], Jiahui Zhang [a], Ze Yin [a], Fasheng Li [a,*]

[a] College of Medical Laboratory, Dalian Medical University, Dalian 116044, China

*Correspondence to: Fasheng Li (E-mail: lifasheng@dmu.edu.cn), Tel: +86-411-86110391

Supplementary Material S1 . Western blot

RIPA buffer was used to extract total proteins from the cells. The extracted proteins were quantified using a BCA kit and separated on an SDS-PAGE gel. The separated proteins were then transferred to a PVDF membrane, and non-specific sites were blocked using 5% skim milk.The membrane was incubated with primary antibodies and left to stand at 4°C overnight. The next day, the membrane was washed three times with TBST and then incubated with secondary antibodies for 2 hours. Thereafter, the membrane was washed thrice with TBST, and protein bands were detected using enhanced chemiluminescence. For in vivo experiments, mouse brain tissues were washed with PBS at 4°C and homogenised in a glass homogeniser on ice. Subsequently, RIPA buffer containing 1% protease and phosphatase inhibitors was added to lyse the tissues for protein extraction. The tissue samples were then incubated at 4℃ for 30 min, followed by centrifugation at 14,000 r/min for 15 min. Total proteins were obtained by absorbing the supernatant. The

concentration was measured by the BCA method. The levels of relevant proteins were analysed using the ImageJ software.

Supplementary Material S2. qRT-PCR

For real-time reverse transcription polymerase chain reaction (qRT-PCR), cDNA was synthesised using the PrimeScriptTM RT Reagent Kit with gDNA eraser according to the manufacturer's instructions. Thereafter, qPCR was performed using SYBR Premix Ex Taq II (TliRNaseH Plus) according to the manufacturer's instructions.

Reagent component	Addition dose
Total RNA/mRNA	0.1ng-5μg
Random Primer(N9) (0.1μg/μL)	1μL
2×ES Reaction Mix	10μL
EasyScript® RT/RI Enzyme Mix	1μL
gDNA Remover	1μL
RNase-free Water	Variable
Total volume	20μL

Reagent component	Addition dose
Template	Variable
Forward Primer(10μM)	0.4μL
Reverse Primer(10μM)	0.4μL
2×TransStart ® Top Green Qpcr SuperMix	10μL

Nuclease-free Water	Variable
Total volume	20μL

Gene	sequences
GAPDH (mouse)	F: AAGCTCATTTCCTGGTATGACAA<br>R: CTTACTCCTTGGAGGCCATGT
Lnc Gm16410 (mouse)	F: GCCCATCAGTTGTGCCTTCT<br>R: ACATCCCGGCTAGTTGTTCTT
TNF-α (mouse)	F: CGATGGGTTGTACCTTGTCTACT<br>R: GGAGGTTGACTTTCTCCTGGTAT
IL-1β (mouse)	F: TCCAGGATGAGGACATGAGCAC<br>R: GAACGTCACACACCAGCAGGTTA
